# Supplementary material for: Phenazine production promotes antibiotic tolerance and metabolic heterogeneity in Pseudomonas aeruginosa biofilms
Source: Nat Commun. 2019 Feb 15;10:762. doi: 10.1038/s41467-019-08733-w (PMC6377615; doi:10.1038/s41467-019-08733-w)
Supplement: Supplementary file 1 — Supplementary Information [file 41467_2019_8733_MOESM1_ESM.pdf]

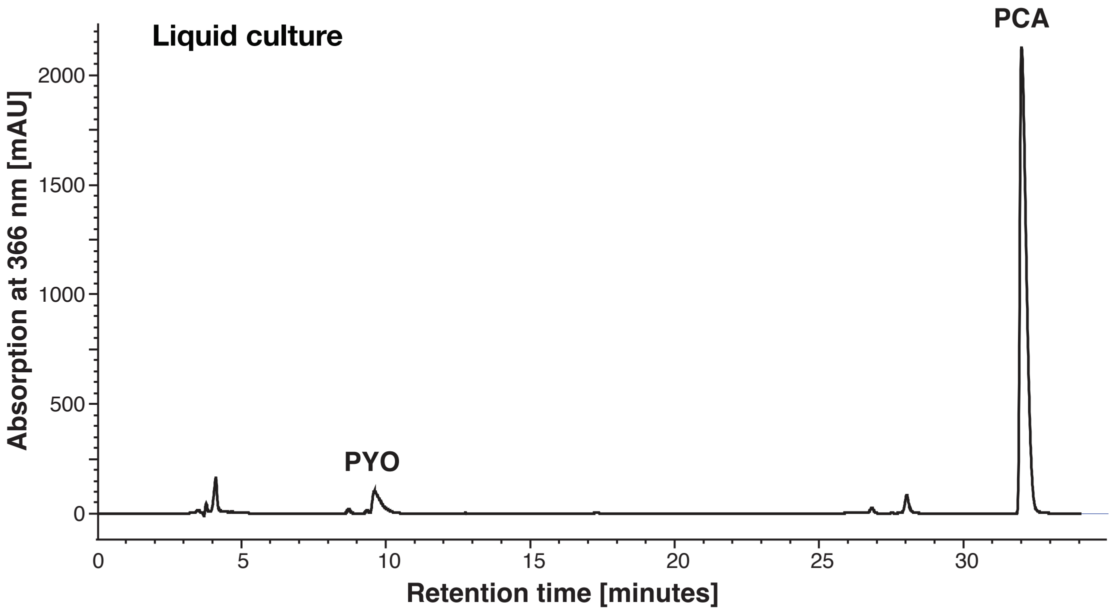


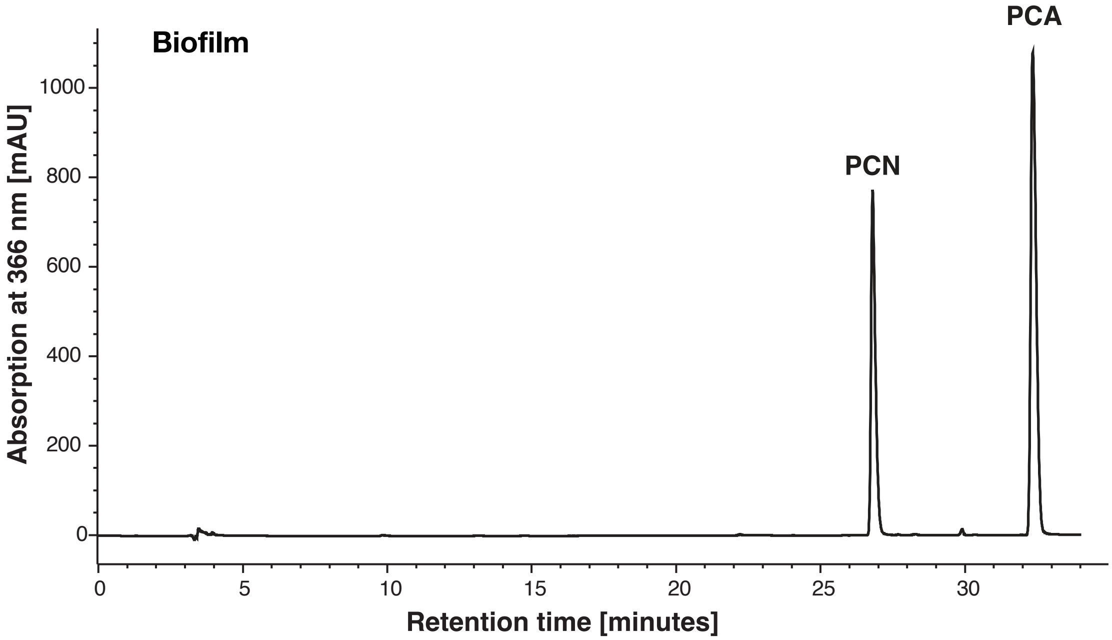


**Supplementary Figure 1. Representative chromatograms showing phenazines produced by *P. aeruginosa* PA14 liquid cultures and biofilms.** Liquid cultures were grown in 50 ml MOPS-glucose in a 250-ml Erlenmeyer flask) with shaking at 250 rpm for 16 hours before supernatant was collected for HPLC analysis. Colony biofilms were each grown for 89 hours on 6 ml MOPS-glucose with 1% agar. Phenazines were extracted from a combined homogenate of the biofilm and agar-solidified medium. Phenazine-1-carboxamide (PCN) was not detected in liquid-culture samples while pyocyanin (PYO) was not detected in samples from biofilms. PCA, phenazine-1-carboxylic acid. Chromatograms are representative of five biological replicates for liquid cultures and seven biological replicates for biofilms.

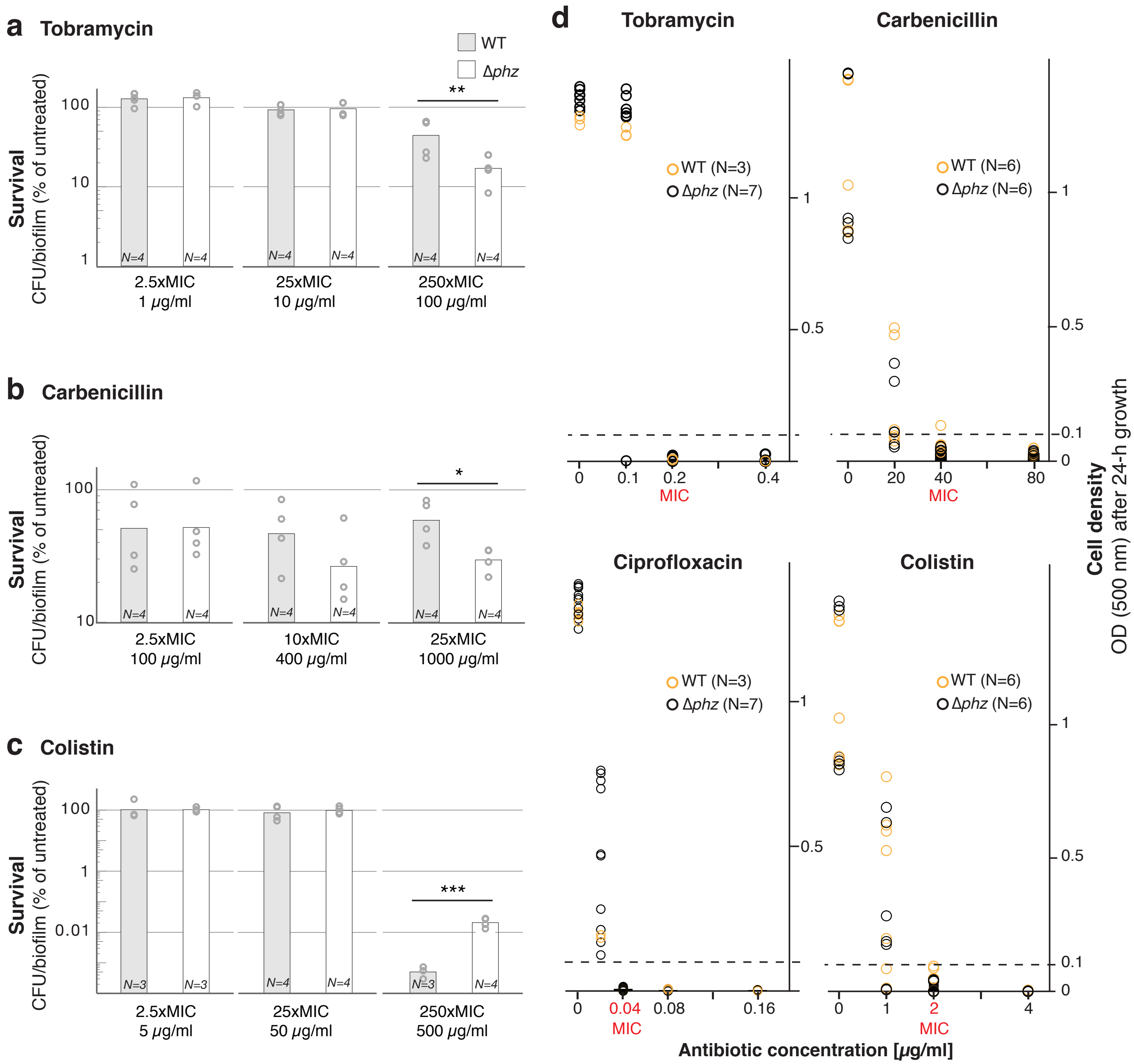


**Supplementary Figure 2. a-c. Survival of PA14 WT and Δ*phz* cells after exposure to antibiotics during growth in a biofilm.** Circles show biological replicates, bars indicate the mean. Significant p-values are indicated and based on unpaired, two-sided t-tests (*, $p \leq 0.05$; **, $p \leq 0.01$; ***, $p \leq 0.001$). While phenazine production antagonizes killing by tobramycin and carbenicillin at higher concentrations, colistin and phenazines show a synergistic killing effect. **d**. Minimum inhibitory concentration (MIC) of ciprofloxacin, tobramycin, colistin and carbenicillin deduced from growth in MOPS-glucose. Cultures were grown in 96-well plates with shaking for 24 hours. The MIC for each antibiotic is the lowest tested concentration at which the average maximal OD is less than 0.1 (indicated by a dashed line). Circles show biological replicates.

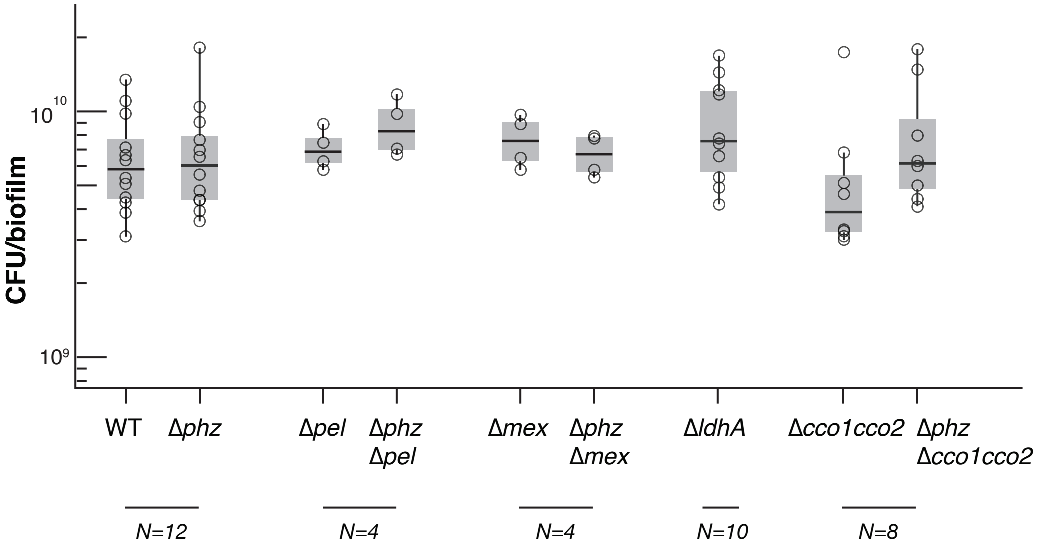


**Supplementary Figure 3. Growth in the absence of ciprofloxacin.** Colonies were grown for 65 hours on MOPS-glucose + 1% agar and then transferred to a fresh plate of medium and incubated for 24 additional hours. Circles show biological replicates. Boxplots indicate the median and the first and third quartile. A Kruskal-Wallis test was carried out to test the effect of strain identity on the number of CFUs per biofilm. There was no statistically significant effect of strain identity [$\chi^2(9)=10.796$, p=0.29]. The center line of the boxplot shows the median, the lower and upper hinges correspond to the first and third quartiles, and the whiskers extend to the most extreme points, limited to 1.5 times the interquartile range.

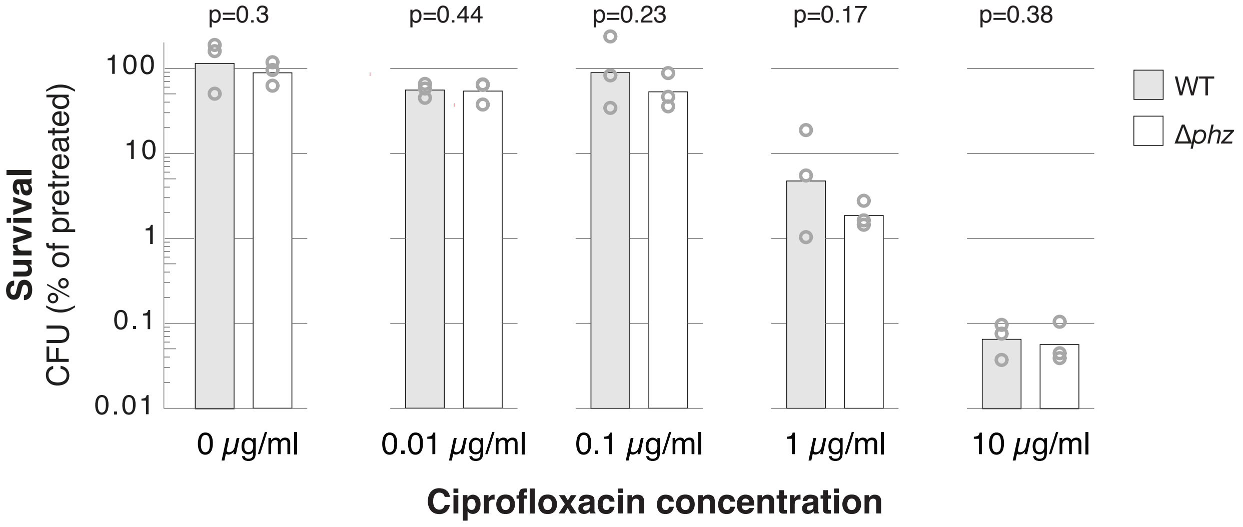


**Supplementary Figure 4. Survival of cells from stationary-phase liquid cultures after exposure to ciprofloxacin.** Replicate cultures (each grown in 50 ml MOPS-glucose medium in a 250-ml flask) were started from the same preculture and grown for 16 hours to stationary phase. Ciprofloxacin was then added at the indicated concentration. Samples were taken for CFU quantification shortly before antibiotic addition and after four hours of incubation in antibiotic. Circles show biological replicates (N=3), bars indicate the mean. P-values are based on unpaired one-sided t-tests.

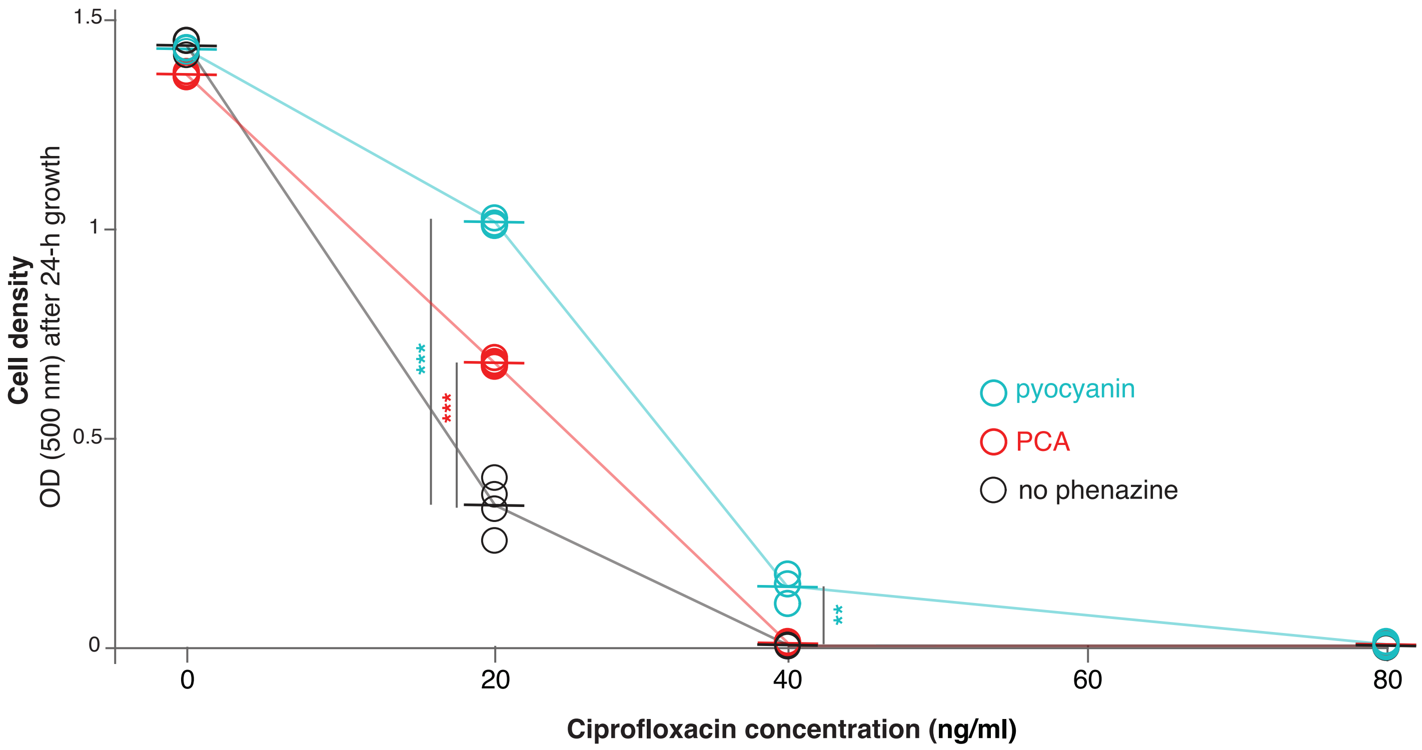


**Supplementary Figure 5. Added phenazines can enhance the growth of Δ*phz* in the presence of ciprofloxacin.** Phenazine-1-carboxylic acid (PCA) or pyocyanin was added to the medium at a final concentration of 300 μM at the onset of the experiment. Liquid cultures were grown in a 96-well plate for 24 h. Circles show biological replicates (N=4 for PCA and no phenazine, N=3 for pyocyanin), crossbars indicate the mean. P values are based on unpaired one-sided t-tests (**, p ≤ 0.01; ***. p ≤ 0.001).

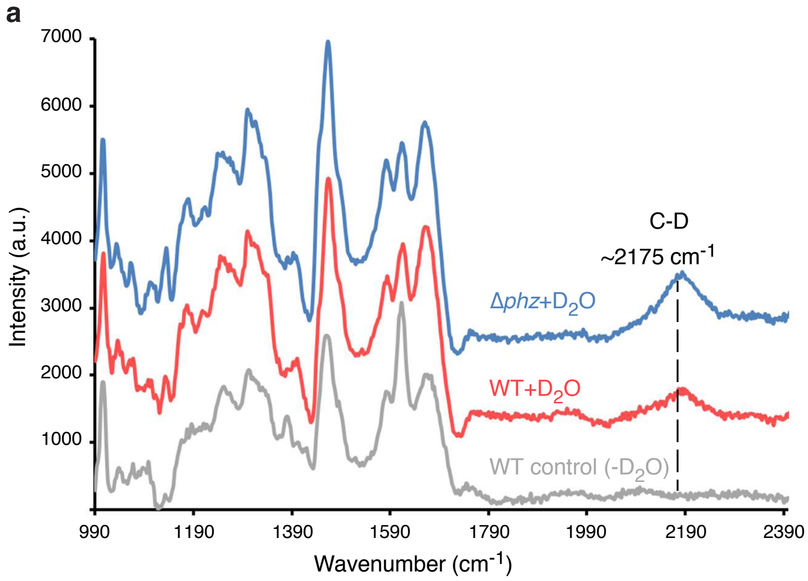


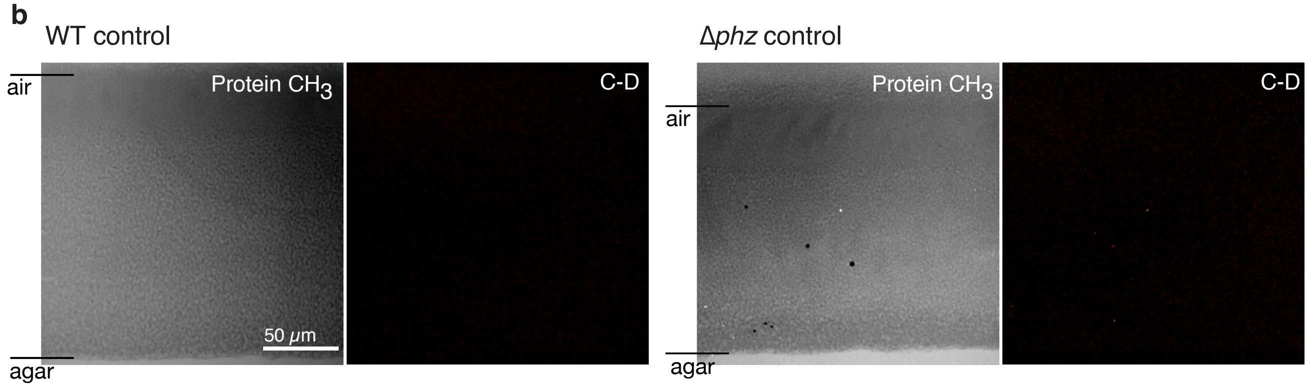


**Supplementary Figure 6. Raman spectra of biofilm thin sections with and without $D_2O$ metabolic labeling. a.** Clear C-D peaks (dashed line) are observed in the cell spectral-silent regions in both WT and Δ*phz* biofilms. **b.** In both strains without deuterium labeling, a negligible signal is observed in the SRS images for background-free detection.

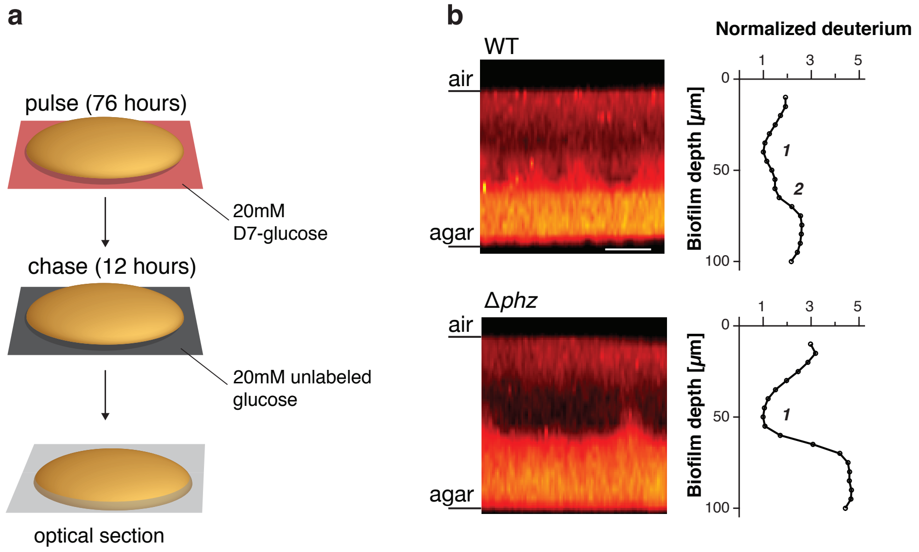


**Supplementary Figure 7. Measurement of metabolic activity in colony biofilms.** Colonies were grown with D7-glucose and then transferred to unlabeled glucose for a 12-hour period before imaging. **a.** Experimental design for D7-glucose labeling scheme. **b.** Distribution of deuterium signal in colony biofilm optical sections prepared as described in panel a. Image and data show one biological replicate representative of N=3. In this labeling regime, metabolic activity is indicated by deuterium depletion and visible as dark zones in the biofilm images. As in the reverse labeling regime (i.e., incorporation of deuterium from $D_2O$), the WT shows two regions of activity (valleys 1 and 2), while Δ*phz* shows one broad region of activity. Deuterium signal in data plots is corrected for light scattering using the protein channel and normalized to the minimal signal in valley 1. Scale bar is 25 μm.

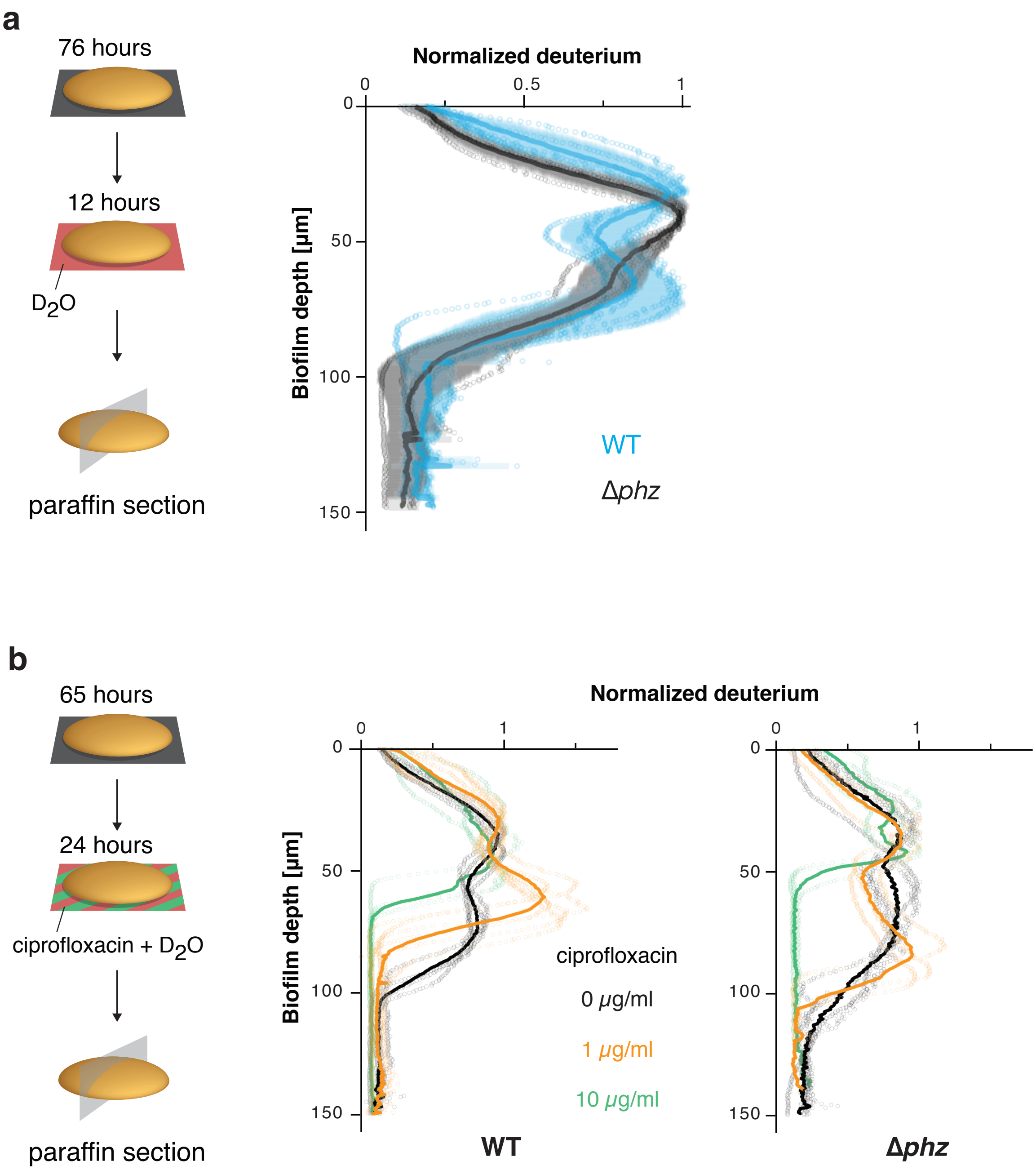


**Supplementary Figure 8. Overview of thin section results from all replicates of experiments described in Figure 3. a.** Deuterium signal in WT (blue, N=6) and Δ*phz* (black, N=5) biofilm sections after incubation as depicted in the schematic shown at left. **b.** Deuterium signal in WT and Δ*phz* after incubation as depicted in the schematic shown at left. N values for each strain/condition ranged from three to six. The signal is normalized to the maximum signal reached in the peak closest to the air interface. Solid line shows average, dots show biological replicates, and standard deviation is indicated by shading.

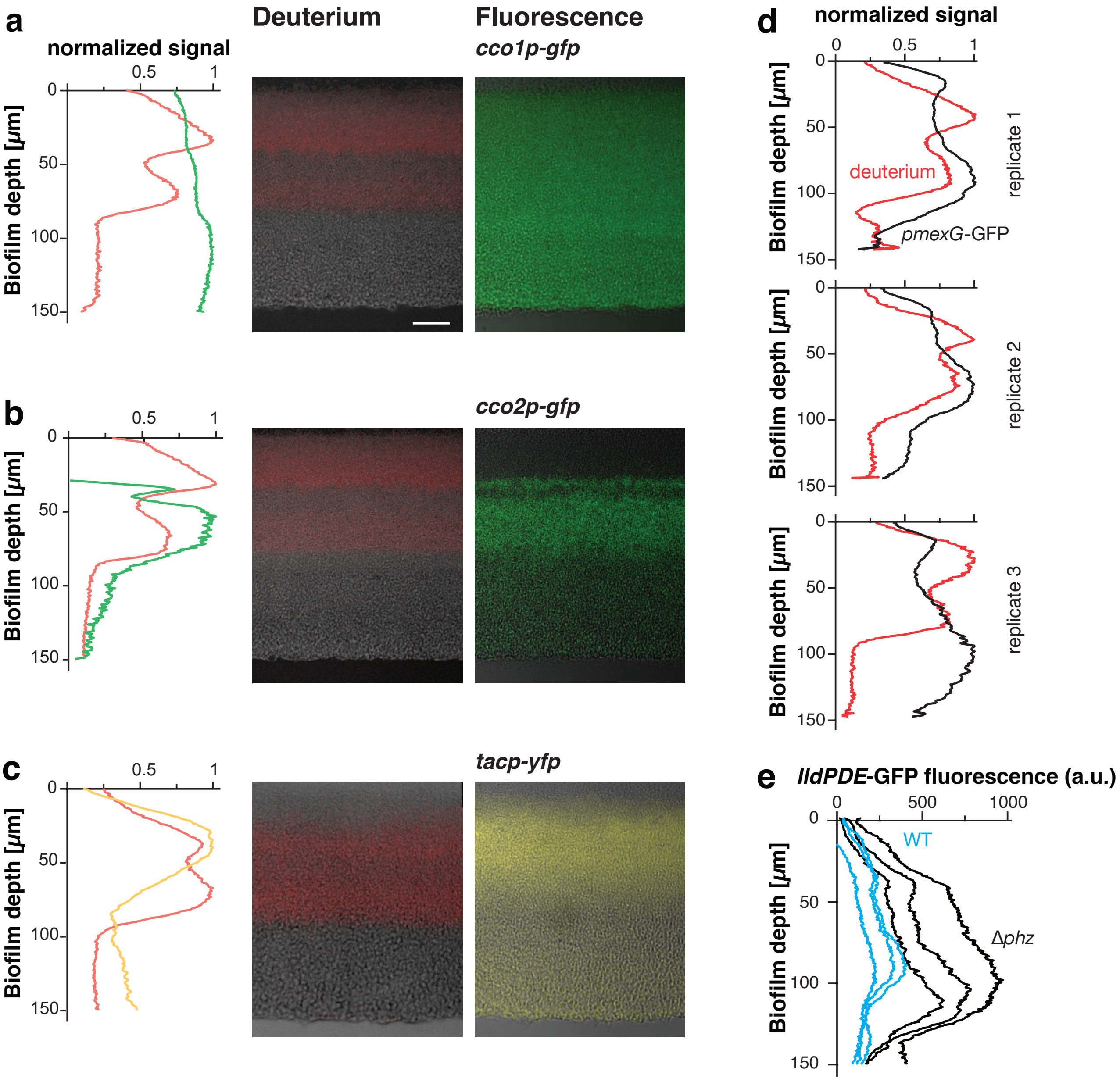


**Supplementary Figure 9. Distribution of metabolic activity and *cco* and *mexG* expression.** Distribution of metabolic activity **w**as compared to expression of the *cco1* and *cco2* operons (encoding *cbb*$_3$-type terminal oxidases) (**a, b**), expression from a constitutive promoter (**c**) and expression driven by the transcription factor *SoxR* (in a *mexGp-gfp* reporter strain) (**d**) in WT biofilms. For **a-c**, images and data plots show paraffin sections of one biological replicate representative of N=3 for a, b and 4 for c. Colonies were grown for three days and then incubated for 12h on medium containing 50% $D_2O$. Deuterium and fluorescence signals in data plots are normalized to the maximum for each sample and type of signal. Scale bar is 25 µm. Panel **e** shows all replicates of experiments described in **Figure 4c** of *lldPDE* expression in WT (blue, N=3) and Δ*phz* (black, N=3).

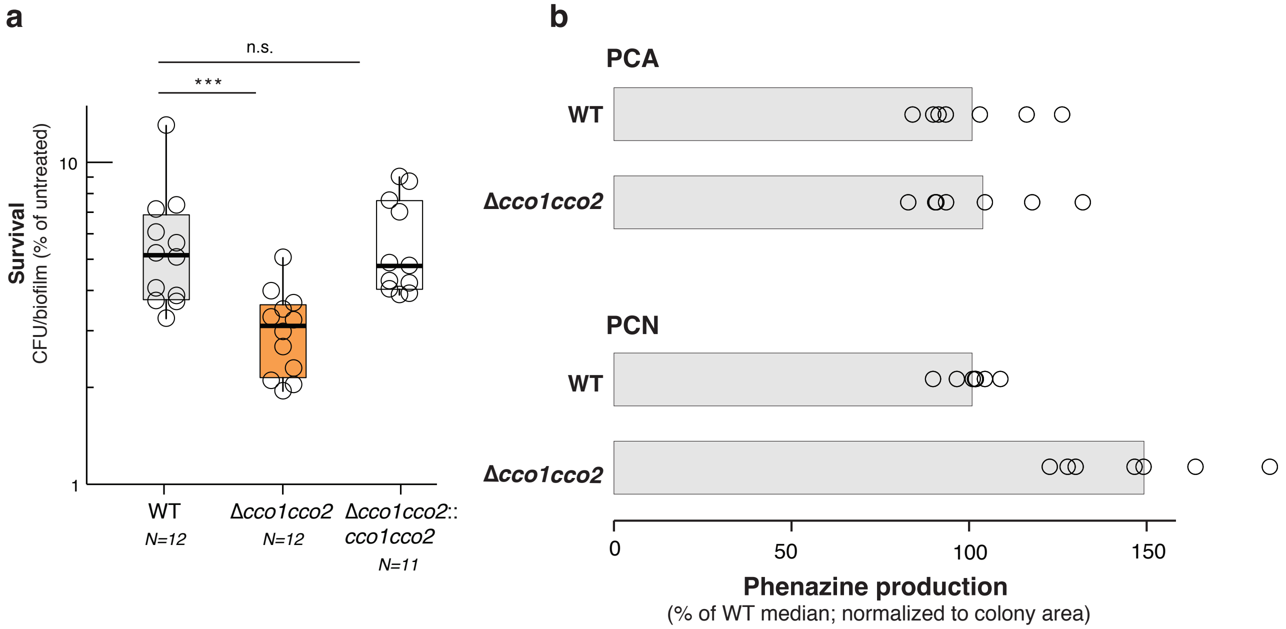


**Supplementary Figure 10. Control experiments for the deletion mutant Δ*cco1cco2* confirm the importance of this locus for the phenazine-protective effect. a.** Ciprofloxacin (100 µg/mL) tolerance observed for cells from biofilms formed by the Δ*cco1cco2* complementation strain (Δ*cco1cco2*::*cco1cco2*) as compared to WT and Δ*cco1cco2*. P value is based on an unpaired two-sided t-test between strain pairs as indicated (n.s., not significant; ***, $p \leq 0.001$). The center line of the boxplot shows the median, the lower and upper hinges correspond to the first and third quartiles, and the whiskers extend to the most extreme points, limited to 1.5 times the interquartile range. **b.** Phenazine production by Δ*cco1cco2* biofilms is similar to phenazine production by WT biofilms. Circles show biological replicates (N=7 for WT, N=8 for Δ*cco1cco2*), bars indicate mean of replicates. Biofilms were grown for four days before sampling. Phenazines were extracted from both the agar-solidified medium and the biofilm for each sample.

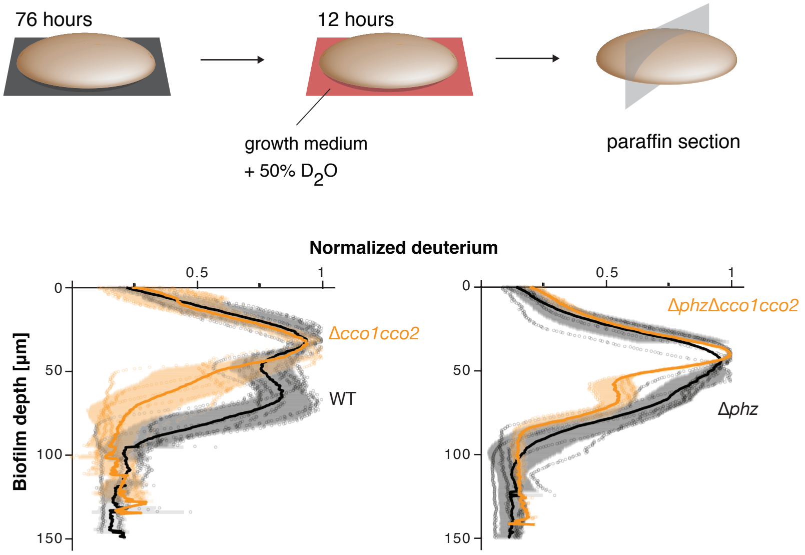


**Supplementary Figure 11. The distribution of metabolic activity across biofilm depth is altered by removal of the Cco terminal oxidases.** Profiles of metabolic activity, as indicated by deuterium uptake, are shown for Δ*cco1cco2* mutants in phenazine-producing (WT) and phenazine-null (Δ*phz*) backgrounds. Colony biofilms were incubated according to the schematic shown and activity profiles are plotted in orange for Δ*cco1cco2* mutants. For comparison, the parent strains are plotted in black (same data as in **Supplementary Figure 8a**). The signal is normalized to the maximum signal reached in the peak closest to the air interface. The solid line shows average, dots show biological replicates (N=5 for Δ*cco1cco2*, N=3 for Δ*phz*Δ*cco1cco2*), and standard deviation is indicated by shading.

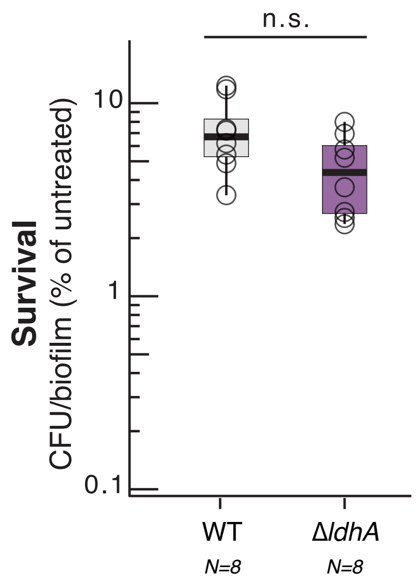


**Supplementary Figure 12. Deletion of *ldhA* does not significantly affect survival of cells in colony biofilms exposed to 100 µg/ml ciprofloxacin.** The lack of significance was determined by an unpaired two-sided t-test (n.s., not significant). The center line of the boxplot shows the median, the lower and upper hinges correspond to the first and third quartiles, and the whiskers extend to the most extreme points, limited to 1.5 times the interquartile range.

**Supplementary Table 1. Bacterial and fungal strains used in this study.**

| Number | Strain | Description | Source |
|---|---|---|---|
| *Pseudomonas aeruginosa* PA14 strains | | | |
| | PA14 (WT) | Clinical isolate UCBPP-PA14 *Pseudomonas aeruginosa* | 1 |
| LD24 | Δ*phz* | PA14 with deletions in *phzA1-G1* and *phzA2-G2* operons | 2 |
| LD2329 | Δ*mexGHI-opmD* Δ*mexVW*Δ*mexPQ-opmE* | PA14 with deletions in PA14_09500-PA14_09540, PA14_56880 -PA14_56890 and PA14_18760-PA14_18790 | This study |
| LD2330 | Δ*phz*Δ*mexGHI-opmD* Δ*mexVW*Δ*mexPQ-opmE* | LD24 (Δ*phz*) with deletions in PA14_09500-PA14_09540, PA14_56880 -PA14_56890 and PA14_18760-PA14_18790 | This study |
| LD82 | Δ*pel* | PA14 with deletions in *pelB-G* | 3 |
| LD83 | Δ*phz*Δ*pel* | PA14Δ*phz* with deletions in *pelB-G* | 3 |
| LD1933 | Δ*cco1cco2* | PA14 with deletions in PA14_44340-PA14_44400 | 4 |
| LD3182 | Δ*cco1cco2*::*cco1cco2* | LD1933 (Δ*cco1cco2*) with *cco1cco2* complemented back into the site of deletion. Made by mating pLD3175 into LD1933. | This study |
| LD1938 | Δ*phz*Δ*cco1cco2* | LD24 (Δ*phz*) with deletions in PA14_44340-PA14_44400 | This study |
| LD2729 | Δ*ldhA* | PA14 with deletion in PA14_52270 | 5 |
| LD2820 | MCS-gfp | PA14 with promoterless *gfp* expression reporter | 4 |
| LD2784 | *cco1pr-gfp* | PA14 with promoter of *cco1* operon driving *gfp*-expression | 4 |
| LD2786 | *cco2pr-gfp* | PA14 with promoter of *cco2* operon driving *gfp*-expression | 4 |
| LD2798 | P*lldPDEpr-gfp* | PA14 with promoter of *lldP* operon driving | 5 |

| | | | |
|---|---|---|---|
| | | *gfp*-expression | |
| LD2799 | Δ*phz*P*lldPDEpr-gfp* | PA14 with promoter of *lldP* operon driving *gfp*-expression in Δ*phz* background | 5 |
| LD36 | *PA1/04/03p*-yfp | PA14 with constitutive tac promoter driving *yfp*-expression | 6 |
| LD808 | P*mexG-gfp* | PA14 with mexG promoter driving gfp expression | This study |
| *Escherichia coli* | | | |
| LD44 | UQ950 | *E. coli* DH5 λpir strain for cloning. $F^-$Δ(*argF- lac*) 169φ80d*lacZ58*(ΔM15) *glnV44*(AS) *rfbD1 gyrA96*($Nal^R$) *recA1 endA1 spoT thi-1 hsdR17 deoR* $\lambda pir^+$ | D. Lies, Caltech |
| LD661 | BW29427 | Donor strain for conjugation; *thrB1004 pro thi rpsL hsdS lacZ* ΔM15RP4-1360 Δ(*araBAD*)*567* Δ*dapA1314::*[*erm pir*(wt)] | W. Metcalf, University of Illinois |
| LD69 | β2155 | Helper strain. thrB1004 pro thi strA hsdsS lacZDM15 (F'lacZDM15 lacIq traΔ36 $proA^+$ $proB^+$ ) ΔdapA:: erm (Ermr)pir::RP4 [::kan (Kmr) from SM10] | 7 |
| LD2901 | S17-1 | $Str^R$, $Tp^R$, $F^-$ RP4-2-Tc::Mu *aphA*::Tn7 *recA* λpir lysogen | 8 |
| *Saccharomyces cerevisiae* | | | |
| LD676 | InvSc | *MATα/MATα leu2/leu2 trp1-289/trp1-289 ura3-52/ura3-52 his3-Δ1/his3-Δ1* | Invitrogen |

**Supplementary Table 2. Plasmids used in this study.**

| Plasmid | Description | Source |
|---|---|---|
| pMQ30 | Yeast-based allelic-exchange vector; *sacB*$^+$, CEN/ARSH, URA3$^+$, $Gm^R$ | 9 |
| pLD2722 | $Gm^R$, $Tet^R$ flanked by Flp recombinase target (FRT) sites to resolve out resistance cassettes | 4 |

| | | |
|---|---|---|
| pFLP2 | Site-specific excision vector with cI857-controlled FLP recombinase encoding sequence, $sacB^+$, $Amp^R$ | 10 |
| pLD2297 | Δ3522 PCR fragment introduced into pMQ30 by gap repair cloning in yeast strain InvSc1. | This study |
| pLD2285 | Δ*mexVW* PCR fragment introduced into pMQ30 by gap repair cloning in yeast strain InvSc1. | This study |
| pLD2273 | Δ*mexAB-oprM* PCR fragment introduced into pMQ30 by gap repair cloning in yeast strain InvSc1. | This study |
| pLD1929 | Δ*cco1cco2* PCR fragment introduced into pMQ30 by gap repair cloning in yeast strain InvSc1. | 4 |
| pLD3175 | *cco1cco2* PCR fragments introduced into pMQ30 by gap repair cloning in yeast strain InvSc1. | This study |
| pLD1599 | *mexG* promoter PCR fragment ligated into pYL122 using HindIII and EcoRI. | This study |

**Supplementary Table 3. Primers used in this study.**

| | |
|---|---|
| Primers for plasmid pLD2297 (used to make Δ3522) | |
| LD1403 | ccaggcaaattctgttttatcagaccgcttctgcgttctgaCCTACAACCAGGCCGATGC |
| LD1404 | ggagaggtatcgtcggtagcGATAAGGGCCGCGACACCTA |
| LD1405 | taggtgtcgcggcccttatcGCTACCGACGATACCTCTCC |
| LD1406 | ggaattgtgagcggataacaatttcacacaggaaacagcAGCGTTCTTTCCACAATAGCC |
| Primers for plasmid pLD2285 (used to make Δ*mexVW*) | |
| LD1411 | ccaggcaaattctgttttatcagaccgcttctgcgttctgaAACGACACTACCGATCCCG |
| LD1412 | aggacgaacagggtgaagagCTGACGGATGGAGTAGAC |
| LD1413 | gtctactccatccgtcagCTCTTCACCCTGTTCGTCCT |
| LD1414 | ggaattgtgagcggataacaatttcacacaggaaacagctGCACTCTGGCTGTCGATCA |
| Primers for plasmid pLD2273 (used to make Δmex*AB-oprM*) | |
| LD1371 | ccaggcaaattctgttttatcagaccgcttctgcgttctgCATCCCAGGAAGTCGAGCTG |
| LD1372 | cacggtctgctggttccaTTCCAGGGTCACGATTCC |
| LD1373 | ggaatcgtgaccctggaaTGGAACCAGCAGACCGTG |

| | |
|---|---|
| LD1374 | ggaattgtgagcggataacaatttcacacaggaaacagctGATGCCCAGGGTTTCGACC |
| Primers for plasmid pLD1929 (used to make Δ*cco1cco2*) | |
| LD725 | ccaggcaaattctgttttatcagaccgcttctgcgttctgatCCCCTCAGAGAAGTCAGTCG |
| LD1063 | gttgcccaggtgttcctgtGGCGGACCACCTTGTAGTTA |
| LD949 | ggaattgtgagcggataacaatttcacacaggaaacagctTGTAGTCGAGGGACTTCTTGC |
| LD1064 | taactacaaggtggtccgccACAGGAACACCTGGGCAAC |

| Primers for plasmid pLD3175 (used to make Δ*cco1cco2*::*cco1cco2*) | |
|---|---|
| LD725 | ccaggcaaattctgttttatcagaccgcttctgcgttctgatCCCCTCAGAGAAGTCAGTCG |
| LD2540 | ACCACGGCATCCATTTCGGTCTTGCCTTTCAGCGAGTC |
| LD2541 | GACTCGCTGAAAGGCAAGACCGAAATGGATGCCGTGGT |
| LD2542 | TTTCGGGATCAGGTGGTAGAGCGAGCCGATGGAGATCAT |
| LD2543 | ATGATCTCCATCGGCTCGCTCTACCACCTGATCCCGAAA |
| LD949 | ggaattgtgagcggataacaatttcacacaggaaacagctTGTAGTCGAGGGACTTCTTGC |

| Primers for plasmid pLD1599 (used to make P*mexG-gfp*) | |
|---|---|
| LD2596 | taccaagcttCTCGTGGCCAACCAGAATAG |
| LD2597 | ttgcgaattcGTCGTTCCTTGTGCTGGTC |

**Supplementary References**


1. Rahme, L. G., Stevens, E. J., Wolfort, S. F., Shao, J., Tompkins, R. G. & Ausubel, F. M. Common virulence factors for bacterial pathogenicity in plants and animals. *Science* **268** (5219), 1899–1902 (1995).
2. Dietrich, L. E. P., Price-Whelan, A., Petersen, A., Whiteley, M. & Newman, D. K. The phenazine pyocyanin is a terminal signalling factor in the quorum sensing network of Pseudomonas aeruginosa. *Mol. Microbiol.* **61** (5), 1308–1321 (2006).
3. Dietrich, L. E. P., Okegbe, C., Price-Whelan, A., Sakhtah, H., Hunter, R. C. & Newman, D. K. Bacterial Community Morphogenesis Is Intimately Linked to the Intracellular Redox State. *J. Bacteriol.* **195** (7), 1371–1380 (2013).
4. Jo, J., Cortez, K. L., Cornell, W. C., Price-Whelan, A. & Dietrich, L. E. An orphan cbb3-type cytochrome oxidase subunit supports Pseudomonas aeruginosa biofilm growth and virulence. *Elife* **6**, doi:10.7554/eLife.30205 (2017).
5. Lin, Y. C., Cornell, W. C., Price-Whelan, A. & Dietrich, L. E. P. The Pseudomonas aeruginosa complement of lactate dehydrogenases enables use of D-and L-lactate and metabolic crossfeeding. *mBio*. **9** (5) e00961-18; doi:10.1128/mBio.00961-18 (2018).
6. Ramos, I., Dietrich, L. E. P., Price-Whelan, A. & Newman, D. K. Phenazines affect biofilm

formation by Pseudomonas aeruginosa in similar ways at various scales. *Res. Microbiol.* **161** (3), 187–191 (2010).
7. Dehio, C. & Meyer, M. Maintenance of broad-host-range incompatibility group P and group Q plasmids and transposition of Tn5 in Bartonella henselae following conjugal plasmid transfer from Escherichia coli. *J. Bacteriol.* **179** (2), 538–540 (1997).
8. Simon, R., Priefer, U. & Pühler, A. A Broad Host Range Mobilization System for In Vivo Genetic Engineering: Transposon Mutagenesis in Gram Negative Bacteria. *Biotechnology* **1**, 784 (1983).
9. Shanks, R. M. Q., Caiazza, N. C., Hinsa, S. M., Toutain, C. M. & O'Toole, G. A. Saccharomyces cerevisiae-based molecular tool kit for manipulation of genes from gram-negative bacteria. *Appl. Environ. Microbiol.* **72** (7), 5027–5036 (2006).
10. Hoang, T. T., Karkhoff-Schweizer, R. R., Kutchma, A. J. & Schweizer, H. P. A broad-host-range Flp-FRT recombination system for site-specific excision of chromosomally-located DNA sequences: application for isolation of unmarked Pseudomonas aeruginosa mutants. *Gene* **212** (1), 77–86 (1998).
